# Clinical characteristics and outcomes of children with Kawasaki disease combined with sepsis in the pediatric intensive care unit

**DOI:** 10.3389/fcimb.2023.1101428

**Published:** 2023-05-10

**Authors:** Yufan Yang, Jiaotian Huang, Haipeng Yan, Xun Li, Pingping Liu, Wu Zhou, Xinping Zhang, Xiulan Lu, Zhenghui Xiao

**Affiliations:** ^1^Department of Pediatric Intensive Care Unit, Hunan Children’s Hospital, Changsha, Hunan, China; ^2^Pediatrics Research Institute of Hunan Province, Hunan Children’s Hospital, Changsha, Hunan, China

**Keywords:** sepsis, Kawasaki disease, pediatric Care Unit, lymphocyte, children

## Abstract

**Background:**

Kawasaki disease (KD) is a vascular inflammatory disease with unknown pathogenesis. There are few studies on KD combined with sepsis worldwide.

**Purpose:**

To provide valuable data regarding clinical characteristics and outcomes related to pediatric patients with KD combined with sepsis in pediatric intensive care unit (PICU).

**Methods:**

We retrospectively analyzed the clinical data of 44 pediatric patients admitted in PICU at Hunan Children’s Hospital with KD combined with sepsis between January 2018 and July 2021.

**Results:**

Of the 44 pediatric patients (mean age, 28.18 ± 24.28 months), 29 were males and 15 were female. We further divided the 44 patients into two groups: KD combined with severe sepsis (n=19) and KD combined with non-severe sepsis (n=25). There were no significant between-group differences in leukocyte, C-reactive protein, and erythrocyte sedimentation rate. Interleukin-6, interleukin-2, interleukin-4 and procalcitonin in KD with severe sepsis group were significantly higher than those in KD with non-severe sepsis group. And the percentage of suppressor T lymphocyte and natural killer cell in severe sepsis group were significantly higher than those in non-severe group, while the CD4^+^/CD8^+^ T lymphocyte ratio was significantly lower in KD with severe sepsis group than in KD with non-severe sepsis group. All 44 children survived and were successfully treated after intravenous immune globulin (IVIG) combined with antibiotics.

**Conclusion:**

Children who develop with KD combined with sepsis have different degrees of inflammatory response and cellular immunosuppression, and the degree of inflammatory response and cellular immunosuppression is significantly correlated with the severity of the disease.

## 1 Introduction

Kawasaki disease (KD), also known as skin mucosal lymph node syndrome, is an acute, self-limiting systemic vascular inflammation in children. The clinical manifestations of KD are fever, conjunctivitis, oral mucous membrane changes, cervical lymphadenopathy, changes in the peripheral extremities, and polymorphous rash (Sundel, 2015; Agarwal and Agrawal, 2017; Rife and Gedalia, 2020). Systemic inflammatory response syndrome caused by the infection is defined as sepsis. Severe sepsis in critically ill children is associated with high mortality rates (Kaukonen et al., 2015). Recent reports have described KD cases that were complicated with encephalitis and shock (Li et al., 2019; Furui et al., 2020). However, there are indeed some cases that meet diagnostic criteria of both KD and sepsis, and there is few research on them. We analyzed the cases of 44 children with KD combined with sepsis, who were treated in pediatric intensive care unit (PICU) of Hunan Children’s Hospital between January 2018 and July 2021. This study aimed to analyze the clinical data of pediatric patients with KD combined with sepsis in the PICU in order to provide valuable data regarding clinical characteristics, laboratory findings, treatment options and outcomes related to these pediatric patients.

## 2 Patients and methods

### 2.1 Patients

This was a single-center, retrospective study of the children with KD combined with sepsis, who were treated at the PICU in our hospital (the Hunan Children’s Hospital, Changsha, China) between January 2018 and July 2021. Forty-four children were diagnosed with KD combined with sepsis at the hospital according to the standard diagnostic criteria (Singer et al., 2016; Kobayashi et al., 2020). The exclusion criteria were: patients with other cardiovascular, hypotension, or hypertension diseases, and primary disease associated with tumors, hematological diseases, congenital malformations, genetic metabolic diseases, primary myocarditis, primary diseases of major organs. We divided the 44 patients into the following two groups based on the severity of sepsis: KD combined with severe sepsis and KD combined with non-severe sepsis.

### 2.2 Definition of sepsis, severe sepsis, and diagnosis of KD

In 2005, definitions and criteria for sepsis and severe sepsis in children based on prevailing views of adult sepsis at the time with modifications for physiology based on age and maturational considerations was published by the International Pediatric Sepsis Consensus Conference (Goldstein et al., 2005). In 2016, new adult definitions and criteria for sepsis and severe sepsis were published (Sepsis-3) (Singer et al., 2016). The term “severe sepsis” was replaced by this new definition of sepsis in 2016. Although application of Sepsis-3 to children has been attempted (Matics and Sanchez-Pinto, 2017; Schlapbach et al., 2018), formal revisions to the 2005 pediatric sepsis definitions remain pending (Schlapbach and Kissoon, 2018). Therefore, the majority of studies used to establish evidence for these guidelines referred to the 2005 guideline. As a consequence, we defined the sepsis and severe sepsis based on the definitions and criteria published by International Pediatric Sepsis Consensus Conference in 2005 (Goldstein et al., 2005), Severe sepsis is defined as sepsis plus one of the following: cardiovascular organ dysfunction or acute respiratory distress syndrome or two or more other organ dysfunctions (Goldstein et al., 2005; Weiss et al., 2020). Also, we defined the diagnosis of KD based on the Revision of diagnostic guidelines for Kawasaki disease (6^th^ revised edition) (Kobayashi et al., 2020), and both incomplete KD and complete KD were included in this study.

### 2.3 Clinical data, laboratory data, and echocardiography

Clinical data of gender, age, clinical features and outcomes were collected by us. Also, laboratory results prior to intravenous immunoglobulin (IVIG) administration were collected. Laboratory data included those related to white blood cell count (WBC), C-reactive protein (CRP), erythrocyte sedimentation rate (ESR), procalcitonin (PCT), liver function and kidney function.

Serum cytokine determination was performed in our hospital laboratory. All patients were tested for the following cytokines: interleukin-2 (IL-2), interleukin-4 (IL-4), interleukin-6 (IL-6), interleukin-10 (IL-10), tumor necrosis factor alpha (TNF-α), and interferon gamma (IFN-γ) at admission. Echocardiography was also performed for all patients. And the lymphocyte subgroup analysis was performed at the admission.

### 2.4 Statistical analyses

Two-sample t-test and the Mann-Whitney U test were used to assess between-group differences in the continuous variables. Proportions were compared using Fisher’s exact test and Pearson’s chi-square test. The measurement data are expressed as the mean ± standard deviation (*x* ± s). The statistical analyses were performed using SPSS version 20.0 (IBM, Armonk, NY, USA). Statistical significance was defined as two-sided *P* value < 0.05.

## 3 Results

### 3.1 Clinical features of patients with KD combined with sepsis

The patients (n=44) were divided into two groups: KD combined with severe sepsis and KD combined with non-severe sepsis. The patients with KD combined with severe sepsis were older (36.58 ± 27.51 vs 21.84 ± 19.75 months, *P*<0.05) than those with KD combined with non-severe sepsis. There were no significant differences in sex distribution, clinical KD findings such as conjunctivitis, oral mucous membrane changes, cervical lymphadenopathy, and polymorphous rash between two groups (all *P*> 0.05) (**Table 1**). Echocardiography showed that the diameter of left main coronary artery in KD combined with severe sepsis group was significantly larger than that in KD combined with non-severe sepsis group (2.87 ± 0.75 vs 2.33 ± 0.52 *P*<0.05) (**Table 1**). There is only one patient developing with myocardial injury in Kawasaki disease combined with non-severe sepsis, while there are four patients developing with myocardial injury in Kawasaki disease combined with severe sepsis.

**Table 1:** Clinical features of patients.

	KD with severe sepsis (n=19)	KD with non-severe sepsis (n=25)	*P* value
Age (months) Mean ± SD	36.58 ± 27.51	21.84 ± 19.75	0.044^a^
Female gender No. (%)	9 (47)	6(25)	0.105^b^
Conjunctivitis, No. (%)	12(63)	16(64)	0.95^b^
Oral mucous membrane changes, No.(%)	18(95)	23(92)	0.721^b^
Cervical lymphadenopathy, No.(%)	15(79)	21(84)	0.667^b^
Changes in the peripheral extremity, No.(%)	13(68)	16(64)	0.759^b^
Polymorphous rash, No.(%)	12(63)	18(72)	0.533^b^
Diameter of left main coronary artery (mm)	2.87 ± 0.75	2.33 ± 0.52	0.002^c^
Diameter of right coronary artery (mm)	2.38 ± 0.81	2.04 ± 0.69	0.087^c^

a Two-sample t test.

b Pearson chi square test.

c Manne-Whitney U test.

### 3.2 Laboratory data

The creatinine (43.11 ± 27.46 vs 26.29 ± 6.73 μmol/L), and PCT (12.91 ± 22.94 vs 6.50 ± 10.59 mg/L) were significantly higher in the KD combined with severe sepsis group than in the KD combined with non-severe sepsis group (all *P*<0.05). And the platelet count (248.21 ± 138.39 vs 358.52 ± 183.21×10^9^/L) were significantly lower in the KD combined with severe sepsis group than in the KD combined with non-severe sepsis group (*P*<0.05) (**Table 2**).

**Table 2:** Laboratory data of patients.

	KD with severe sepsis (n=19)	KD with non-severe sepsis (n=25)	*P* value
WBC count (×10^9^/L)	23.41 ± 7.32	22.44 ± 16.44	0.207^c^
Platelet count (× 10^9^/L)	248.21 ± 138.39	358.52 ± 183.21	0.034^a^
ESR (mm/h)	31.32 ± 8.93	37.48 ± 21.65	0.722^c^
CRP (mg/L)	121.71 ± 51.00	112.02 ± 78.08	0.292^c^
ALT (U/L)	112.63 ± 141.40	120.10 ± 189.72	0.414^c^
AST (U/L)	67.86 ± 79.64	78.56 ± 129.47	0.878^c^
Creatinine(μmol/L)	43.11 ± 27.46	26.29 ± 6.73	0.018^c^
BUN(mmol/L)	5.26 ± 3.41	3.42 ± 1.51	0.056^c^
CK-MB(U/L)	14.29 ± 11.96	14.52 ± 12.25	0.67^c^
PCT(ng/ml)	12.91 ± 22.94	6.50 ± 10.59	0.032^c^
D-dimer(mg/L)	5.57 ± 7.96	4.22 ± 6.75	0.095^c^
Albumin (g/L)	31.71 ± 5.93	34.84 ± 5.14	0.082^c^

a Two-sample t test.

c Manne-Whitney U test.

Significantly higher levels of serum IL-6 (404.54 ± 543.38 vs 185.11 ± 242.73 pg/ml), IL-2 (5.30 ± 2.03 vs 4.11 ± 1.67 pg/ml), and IL-4 (4.76 ± 1.31 vs 3.34 ± 1.37 pg/ml) were found in patients with KD combined with severe sepsis (all *P*<0.05, respectively). There were no significant between-group differences in TNF-α and IFN-γ levels (all *P*>0.05) (**Table 3**). However, in this study, IL-2 and IL-4 levels were in normal range, so the increase in IL-6 may be a predictor of KD combined with severe sepsis.

**Table 3:** Serum cytokine of patients.

Cytokine(pg/ml)	KD with severe sepsis (n=19)	KD with non-severe sepsis (n=25)	*P* value
IL-2	5.30 ± 2.03	4.11 ± 1.67	0.037^a^
IL-4	4.76 ± 1.31	3.34 ± 1.37	0.001^c^
IL-6	404.54 ± 543.38	185.11 ± 242.73	0.002^c^
IL-10	108.21 ± 118.95	39.42 ± 42.59	0.132^c^
TNF-ɑ	4.12 ± 2.16	4.04 ± 1.88	0.802^c^
IFN-γ	12.12 ± 8.62	10.26 ± 6.01	0.627^c^

a Two-sample t test.

c Manne-Whitney U test.

### 3.3 Lymphocyte analysis

**Figure 1:**
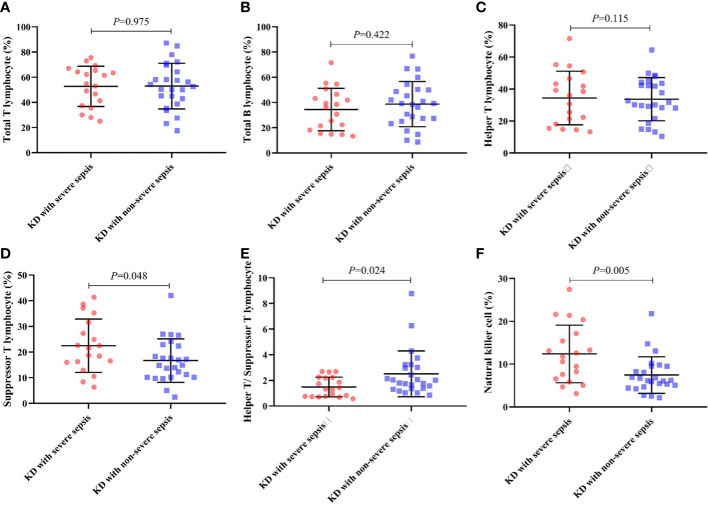
Percentage of lymphocyte subgroup type in each group. **(A)** Total T lymphocyte, **(B)** Total B lymphocyte, **(C)** Helper T lymphocyte, **(D)** Suppressor T lymphocyte, **(E)** CD4^+^/CD8^+^ ratio, **(F)** NK cell.

And during the lymphocyte subgroup type analysis, the percentage of suppressor T-lymphocyte (CD8^+^ T-lymphocyte) (22.47 ± 10.40 vs 16.69 ± 8.45) and natural killer (NK) cell (12.38 ± 6.74 vs 7.44 ± 4.28) in KD with severe sepsis group were significantly higher than those in KD with non-severe group, while the CD4^+^/CD8^+^ T-lymphocyte ratio (1.49 ± 0.77 vs 2.51 ± 1.78) were significantly lower in severe group than in non-severe group (all *P*< 0.05, respectively) (**Table 4**; **Figure 1**).

**Table 4:** Lymphocyte subgroup type in patients.

	KD with severe sepsis (n=19)	KD with non-severe sepsis (n=25)	*P* value
Total T lymphocytes (%)	52.77 ± 15.96	52.94 ± 18.16	0.975^a^
Total B lymphocyte(%)	34.41 ± 15.96	38.72 ± 17.93	0.422^a^
Helper T lymphocyte (CD4^+^ T lymphocyte) (%)	27.71 ± 10.00	33.65 ± 13.50	0.115^a^
Suppressor T lymphocyte (CD8^+^ T lymphocyte) (%)	22.47 ± 10.40	16.69 ± 8.45	0.048^a^
Natural killer cell (%)	12.38 ± 6.74	7.44 ± 4.28	0.005^a^
CD4^+^ T/CD8^+^ T lymphocyte	1.49 ± 0.77	2.51 ± 1.78	0.024^a^

a Two-sample t test.

### 3.4 Information of the proven infection

Also, we found the information of proven infection in patients with KD combined with sepsis. In KD with severe sepsis group, 11 patients were diagnosed with mycoplasma pneumonia, 3 patients were found infected with group A streptococcal, 1 patient was found infected with *Staphylococcus haemolyticus*, 2 patients were found infected with adenovirus, 1 patient was found infected with influenza A virus and 1 patient was found infected with klebsiella pneumoniae. In KD with non-severe sepsis group, 21 patients were diagnosed with mycoplasma pneumonia, 1 patient was found infected with influenza B virus, 1 patient was found infected with cytomegalovirus, 1 patient was found infected with *Haemophilus influenzae* and 1 patient was found infected with *Staphylococcus hominis*.

### 3.5 Treatment and outcomes

In 44 patients, 10 patients were diagnosed with Kawasaki disease shock syndrome (KDSS), no patients were diagnosed with macrophage activation syndrome (MAS). All 44 patients received conventional therapy with aspirin and IVIG. Antibiotic treatment was also used according to bacterial culture or empirically if culture-negative. The length of stay at the PICU was significantly longer for patients with KD combined with severe sepsis than for patients with KD combined with non-severe sepsis (11 ± 3.42 vs 8.08 ± 3.01 days, *P*<0.05). There were no significant between-group differences in hospitalization duration and left ventricular ejection fraction (all *P*> 0.05) (**Table 4**). No fatalities were observed in our study (**Table 5**).

**Table 5:** Treatment and outcome of patients.

Outcome	KD with severe sepsis (n=19)	KD with non-severe sepsis (n=25)	*P* value
Hospitalized duration (days) mean ± SD	14.95 ± 4.72	13.48 ± 4.37	0.171^c^
PICU duration (days) mean ± SD	11 ± 3.42	8.08 ± 3.01	0.004^c^
Ejection fraction, %,(mean± SD)	0.65 ± 0.1	0.7 ± 0.05	0.056^c^
Death No. (%)	0(0.00)	0(0.00)	1.000^d^

c Manne-Whitney U test.

d Fisher’s exact test.

## 4 Discussion

In previous studies, researchers mainly focused on distinguishing KD and sepsis (Liu et al., 2020; Niu et al., 2021), and the research conducted on KD combined with sepsis was rare. The incidence of KD is reported to be significantly higher in China than in Europe and America (Newburger et al., 2004; Uehara and Belay, 2012; Xie et al., 2020). Thus, a strength of this study is that we assessed a relatively large number of patients with KD combined with sepsis. And this study partially reveals the relationship between KD and sepsis. KD may be a cluster of phenotypes triggered by multiple infectious agents and influenced by various environmental, genetic, and immunologic responses (Leung and Schlievert, 2021). And the sepsis is definitely related to the infection. Consequently, there is relationship between sepsis and Kawasaki disease.

KD predominantly occurs in children below five years of age. Our results are similar to those of previous studies (Rife and Gedalia, 2020). But in our study, the patients with KD combined with severe sepsis were older (36.58 ± 27.51 vs 21.84 ± 19.75 months, *P*<0.05) than those with KD combined with non-severe sepsis. Studies show that the younger the children are, the stronger their innate immunity will be, especially in children below the age of 6 months (Beutler, 2004; Kaur and Secord, 2019). But this study only included a retrospective single-center data, prospective multi-center data needed to be conducted to probe the age of KD combined with sepsis.

In **Table 1**, the Echocardiography showed that the diameter of left main coronary artery in KD combined with severe sepsis group was significantly larger than that in KD combined with non-severe sepsis group (2.87 ± 0.75 vs 2.33 ± 0.52 *P*<0.05). It indicates that coronary artery dilatation is related to inflammatory indicators.

In our study, significantly lower platelet count (248.21 ± 138.39 vs 358.52 ± 183.21×10^9^/L) and higher PCT levels (12.91 ± 22.94 vs 6.50 ± 10.59 mg/L) were observed in patients with KD combined with severe sepsis compared to patients with KD combined with non-severe sepsis (all *P*< 0.05). KD can significantly increase platelet count (Ae et al., 2020), while platelet count in patients with sepsis and animal models show a downward trend (Assinger et al., 2019). This can also explain why in our study, platelets in the patients with KD combined with severe sepsis were normal, but significantly lower than those in patients with KD complicated with non-severe sepsis. PCT is an important parameter for diagnosing and monitoring inflammatory diseases. High PCT concentrations are positively correlated with sepsis severity (Annane et al., 2013). In our study, the PCT were significantly higher in patients with KD combined with severe sepsis than in those with KD combined with non-severe sepsis; this indicates that PCT may be a predictor of KD combined with severe sepsis.

The immune system is activated both in KD and sepsis, resulting in an increase in pro-inflammatory cytokines in the circulation. Therefore, these inflammatory cytokines may cause systemic damage (Wang et al., 2013; Si et al., 2017; Song et al., 2019). IL-6 promotes inflammation, which can lead to multiple organ damage. With elevated IL-6 levels, patients develop hypoalbuminemia (Schmidt-Arras and Rose-John, 2016). Our study showed that the concentration of albumin in blood tended to be lower in patients with KD combined with severe sepsis than in patients with KD combined with non-severe sepsis (*P*=0.082) and may reflect the significantly elevated levels of cytokines, such as IL-6.

For lymphocyte subgroup type analysis, Pablo et al (De Pablo et al., 2012). found that patients with sepsis who died exhibited less NK cell depletion than survivors. This study indicates that NK cells are participants in septic shock because patients who survived have more depletion and expressed less early activation. And it explains the reason that NK cell in KD combined with severe sepsis group were significantly higher than those in KD with non-severe sepsis group. Also, the decrease of the CD4^+^/CD8^+^ T-lymphocyte ratio indicates that the cellular immune function is inhibited, indicating the severity of patients.

All 44 patients received conventional therapy with aspirin and IVIG. Antibiotic treatment was also used according to bacterial culture and experienced medication. Our findings indicate that KD combined with sepsis can be effectively treated with IVIG combined with antibiotics.

## 5 Study limitations

This is a single-center study with relatively small size. But our hospital is a designated hospital for sepsis and Kawasaki disease, and the patients are well representative. So, we think this study is clinically significant. And further multicenter studies are needed to elucidate the relationship between sepsis and KD development.

## 6 Conclusion

Children who develop with KD combined with sepsis have different degrees of inflammatory response and cellular immunosuppression, and the degree of inflammatory response and cellular immunosuppression is significantly correlated with the severity of the disease.

## Data availability statement

The original contributions presented in the study are included in the article/supplementary material. further inquiries can be directed to the corresponding authors.

## Ethics statement

This manuscript has obtained human research ethics approval from Ethics Committee of Hunan Children’s Hospital on October 2021, approval number: HCHLL-2021-91. The authors have conducted the research as a number of project or course approved by the Ethics Committee of Hunan Children’s Hospital.

## Author contributions

YY, ZX, and XiL designed this study. YY wrote the manuscript. JH, HY, XuL, PL, WZ, XZ participated in the data analysis, interpretation, and agree to be accountable for the content of the work. All authors contributed to the article and approved the submitted version.

## References

[B1] Ae R. Abrams J. Y. Maddox R. A. Schonberger L. B. Nakamura Y. Shindo A.. (2020). Platelet count variation and risk for coronary artery abnormalities in Kawasaki disease. Pediatr. Infect. Dis. J. 39 (3), 197–203. doi: 10.1097/INF.0000000000002563 31851145

[B2] Agarwal S. Agrawal D. K. (2017). Kawasaki Disease: etiopathogenesis and novel treatment strategies. Expert Rev. Clin. Immunol. 13 (3), 247–258. doi: 10.1080/1744666X.2017.1232165 27590181 PMC5542821

[B3] Annane D. Maxime V. Faller J. P. Mezher C. Clec’h C. Martel P.. (2013). Procalcitonin levels to guide antibiotic therapy in adults with non-microbiologically proven apparent severe sepsis: a randomised controlled trial. BMJ Open 3 (2), e002186. doi: 10.1136/bmjopen-2012-002186 PMC3586059 23418298

[B4] Assinger A. Schrottmaier W. C. Salzmann M. Rayes J. (2019). Platelets in sepsis: an update on experimental models and clinical data. Front. Immunol. 10, 1687. doi: 10.3389/fimmu.2019.01687 31379873 PMC6650595

[B5] Beutler B. (2004). Innate immunity: an overview. Mol. Immunol. 40 (12), 845–859. doi: 10.1016/j.molimm.2003.10.005 14698223

[B6] De Pablo R. Monserrat J. Torrijos C. Martín M. Prieto A. Alvarez-Mon M. (2012). The predictive role of early activation of natural killer cells in septic shock. Crit. Care 16 (2), 413. doi: 10.1186/cc11204 22405329 PMC3681341

[B7] Furui S. Seki M. Minami T. Goto M. Yamagata T. (2020). A case of Kawasaki disease complicated by acute disseminated encephalitis. Pediatr. Int. 62 (7), 872–873. doi: 10.1111/ped.14234 32588500

[B8] Goldstein B. Giroir B. Randolph A. (2005). Members of the International Consensus Conference on Pediatric Sepsis. International Consensus Conference on Pediatric Sepsis International pediatric sepsis consensus conference: definitions for sepsis and organ dysfunction in pediatrics. Pediatr. Crit. Care Med. 6 (1), 2–8. doi: 10.1097/01.PCC.0000149131.72248.E6 15636651

[B9] Kaukonen K. M. Bailey M. Pilcher D. Cooper D. J. Bellomo R. (2015). Systemic inflammatory response syndrome criteria in defining severe sepsis. N Engl. J. Med. 372 (17), 1629–1638. doi: 10.1056/NEJMoa1415236 25776936

[B10] Kaur B. P. Secord E. (2019). Innate immunity. Pediatr. Clin. North Am. 66 (5), 905–911. doi: 10.1016/j.pcl.2019.06.011 31466680

[B11] Kobayashi T. Ayusawa M. Suzuki H. Abe J. Ito S. Kato T.. (2020). Revision of diagnostic guidelines for Kawasaki disease (6th revised edition). Pediatr. Int. 62 (10), 1135–1138. doi: 10.1111/ped.14326 33001522

[B12] Leung D. Y. M. Schlievert P. M. (2021). Kawasaki Syndrome: role of superantigens revisited. FEBS J. 288 (6), 1771–1777. doi: 10.1111/febs.15512 32770775 PMC7436680

[B13] Li Y. Zheng Q. Zou L. Wu J. Guo L. Teng L.. (2019). Kawasaki Disease shock syndrome: clinical characteristics and possible use of IL-6, IL-10 and IFN-γ as biomarkers for early recognition. Pediatr. Rheumatol Online J. 17 (1), 1. doi: 10.1186/s12969-018-0303-4 30611297 PMC6321686

[B14] Liu X. P. Huang Y. S. Kuo H. C. Xia H. B. Yi-Sun Huang W. D.. (2020). A novel nomogram model for differentiating Kawasaki disease from sepsis. Sci. Rep. 10 (1), 13745. doi: 10.1038/s41598-020-70717-4 32792679 PMC7427092

[B15] Matics T. J. Sanchez-Pinto L. N. (2017). Adaptation and validation of a pediatric sequential organ failure assessment score and evaluation of the sepsis-3 definitions in critically ill children. JAMA Pediatr. 171, e172352. doi: 10.1001/jamapediatrics.2017.2352 28783810 PMC6583375

[B16] Newburger J. W. Takahashi M. Gerber M. A. Gewitz M. H. Tani L. Y. Burns J. C. Diagnosis, treatment, and long-term management of Kawasaki disease: a statement for health professionals from the committee on rheumatic fever, endocarditis and Kawasaki disease, council on cardiovascular disease in the young, American Heart Association Circulation. 2004 110(17), 2747–71. 10.1161/01.CIR.0000145143.19711.78 15505111

[B17] Niu M. M. Jiang Q. Ruan J. W. Liu H. H. Chen W. X. Qiu Z.. (2021). Clinical implications of procalcitonin in Kawasaki disease: a useful candidate for differentiating from sepsis and evaluating IVIG responsiveness. Clin. Exp. Med. 21 (4), 633–643. doi: 10.1007/s10238-021-00709-9 33839960 PMC8036161

[B18] Rife E. Gedalia A. (2020). Kawasaki Disease: an update. Curr. Rheumatol Rep. 22 (10), 75. doi: 10.1007/s11926-020-00941-4 32924089 PMC7487199

[B19] Schlapbach L. J. Kissoon N. (2018). Defning pediatric sepsis. JAMA Pediatr. 172, 312–314. doi: 10.1001/jamapediatrics.2017.5208 29459982

[B20] Schlapbach L. J. Straney L. Bellomo R. MacLaren G. Pilcher D. (2018). Prognostic accuracy of age-adapted SOFA, SIRS, PELOD-2, and qSOFA for in-hospital mortality among children with suspected infection admitted to the intensive care unit. Intensive Care Med. 44 (2), 179–188. doi: 10.1007/s00134-017-5021-8 29256116 PMC5816088

[B21] Schmidt-Arras D. Rose-John S. (2016). IL-6 pathway in the liver: from physiopathology to therapy. J. Hepatol. 64 (6), 1403–1415. doi: 10.1016/j.jhep.2016.02.004 26867490

[B22] Si F. Wu Y. Gao F. Feng S. Liu R. Yi Q. (2017). Relationship between IL-27 and coronary arterial lesions in children with Kawasaki disease. Clin. Exp. Med. 17 (4), 451–457. doi: 10.1007/s10238-017-0451-8 28108813

[B23] Singer M. Deutschman C. S. Seymour C. W. Shankar-Hari M. Annane D. Bauer M.. (2016). The third international consensus defnitions for sepsis and septic shock (Sepsis-3). JAMA 315 (8), 801–810. doi: 10.1001/jama.2016.0287 26903338 PMC4968574

[B24] Song J. Park D. W. Moon S. Cho H. J. Park J. H. Seok H.. (2019). Diagnostic and prognostic value of interleukin-6, pentraxin 3, and procalcitonin levels among sepsis and septic shock patients: a prospective controlled study according to the sepsis-3 definitions. BMC Infect. Dis. 19 (1), 968. doi: 10.1186/s12879-019-4618-7 31718563 PMC6852730

[B25] Sundel R. P. (2015). Kawasaki Disease. Rheum Dis. Clin. North Am. 41 (1), 63–73. doi: 10.1016/j.rdc.2014.09.010 25399940

[B26] Uehara R. Belay E. D. (2012). Epidemiology of Kawasaki disease in Asia, Europe, and the united states. J. Epidemiol. 22 (2), 79–85. doi: 10.2188/jea.JE20110131 22307434 PMC3798585

[B27] Wang Y. Wang W. Gong F. Fu S. Zhang Q. Hu J.. (2013). Evaluation of intravenous immunoglobulin resistance and coronary artery lesions in relation to Th1/Th2 cytokine profiles in patients with Kawasaki disease. Arthritis Rheumatol. 65 (3), 805–814. doi: 10.1002/art.37815 23440694

[B28] Weiss S. L. Peters M. J. Alhazzani W. Agus M. S. D. Flori H. R. Inwald D. P.. (2020). Surviving sepsis campaign international guidelines for the management of septic shock and sepsis-associated organ dysfunction in children. Pediatr. Crit. Care Med. 46 (Suppl 1), 10–67. doi: 10.1007/s00134-019-05878-6 PMC7095013 32030529

[B29] Xie L. P. Yan W. L. Huang M. Huang M. R. Chen S. Huang G. Y.. (2020). Epidemiologic features of Kawasaki disease in shanghai from 2013 through 2017. J. Epidemiol. 30 (10), 429–435. doi: 10.2188/jea.JE20190065 31548437 PMC7492704

